# Retractions in general and internal medicine in a high-profile scientific indexing database: Retratações em medicina geral e interna em um indexador científico de alta visibilidade

**DOI:** 10.1590/1516-3180.2014.00381601

**Published:** 2015-08-21

**Authors:** Renan Moritz Varnier Rodrigues de Almeida, Fernanda Catelani, Aldo José Fontes-Pereira, Nárrima de Souza Gave

**Affiliations:** I PhD. Associate Professor, Biomedical Engineering Program (COPPE), Universidade Federal do Rio de Janeiro (UFRJ), Rio de Janeiro (RJ), Brazil.; II MSc. Doctoral Student, Biomedical Engineering Program (COPPE), Universidade Federal do Rio de Janeiro (UFRJ), Rio de Janeiro, Brazil.; III MSc. Physiotherapist, Physiotherapy Department, Hospital Central do Exército, Rio de Janeiro (RJ), Brazil.

**Keywords:** Databases, Bibliographic, Bibliometrics, Retraction of publication, Scientific misconduct, Journal impact factors

## Abstract

**CONTEXT AND OBJECTIVE:**

Increased frequency of retractions has recently been observed, and retractions are important events that deserve scientific investigation. This study aimed to characterize cases of retraction within general and internal medicine in a high-profile database, with interest in the country of origin of the article and the impact factor (IF) of the journal in which the retraction was made.

**DESIGN AND SETTING:**

This study consisted of reviewing retraction notes in the Thomson-Reuters Web of Knowledge (WoK) indexing database, within general and internal medicine.

**METHODS:**

The retractions were classified as plagiarism/duplication, error, fraud and authorship problems and then aggregated into two categories: “plagiarism/duplication” and “others.” The countries of origin of the articles were dichotomized according to the median of the indicator “citations per paper” (CPP), and the IF was dichotomized according to its median within general and internal medicine, also obtained from the WoK database. These variables were analyzed using contingency tables according to CPP (high versus low), IF (high versus low) and period (1992-2002 versus 2003-2014). The relative risk (RR) and 95% confidence interval (CI) were estimated for plagiarism/duplication.

**RESULTS:**

A total of 86 retraction notes were identified, and retraction reasons were found for 80 of them. The probability that plagiarism/duplication was the reason for retraction was more than three times higher for the low CPP group (RR: 3.4; 95% CI: [1.9-6.2]), and similar results were seen for the IF analysis.

**CONCLUSION:**

The study identified greater incidence of plagiarism/duplication among retractions from countries with lower scientific impact.

## RESUMO

**CONTEXTO E OBJETIVO:**

Recente aumento da frequência de retratações tem sido observado, e retratações são eventos importantes que merecem investigação científica. O objetivo do estudo é caracterizar casos de retratação na área de medicina geral e interna em banco de dados de alta visibilidade, com interesse no país de origem do artigo e no Fator de Impacto (FI) da revista que realizou a retratação.

**TIPO DE ESTUDO E LOCAL:**

O estudo consistiu em revisão das notas de retratação no indexador Web of Knowledge (WoK) Thomson Reuters, na área “Medicina Interna e Geral”.

**MÉTODOS:**

As retratações foram classificadas como: plágio e duplicação, erro, fraude e problemas de autoria e, em seguida, agregadas em duas categorias: “plágio e duplicação” e “outros”. Os países de origem dos artigos foram dicotomizados pela mediana do indicador “citações por artigo” (CPP), e o FI foi dicotomizado por sua mediana para a área de pesquisa “medicina interna e geral”, também obtida a partir do WoK. Essas variáveis foram analisadas em tabela de contingência de acordo com os grupos CPP (alto x baixo), FI (alto x baixo) e período (1992-2002, 2003-2014). Riscos relativos (RR) e intervalos de confiança de 95% (IC 95%) foram estimados para plágio-duplicação.

**RESULTADOS:**

Um total de 86 notas de retratação foi identificado, com razões de retratação disponíveis para 80 delas. A probabilidade de plágio/duplicação como razão para a retração foi mais de três vezes maior para o grupo “baixo CPP” (RR: 3,4; IC 95%: [1,9-6,2]), e resultados semelhantes foram observados para a análise de FI.

**CONCLUSÃO:**

O estudo identificou maior incidência de plágio/duplicação nas retratações de países com menor impacto científico.

## INTRODUCTION

The first recorded scientific retraction (withdrawal of a paper after its publication) apparently dates from 1756.^1^ Although uncommon, increased frequency of such events has recently been observed.^2^ Retractions are considered to be important events that deserve scientific investigation.^3^ The reasons commonly mentioned for their occurrence are fraud, ethical issues in human research and issues relating to scientific communication (plagiarism, self-plagiarism and duplication).^3^^,^^4^^,^^5^^,^^6^

More recently, the association between retractions and scientometric factors such as research field, country and other characteristics of authors and journals has become a matter of interest and debate, with a view towards development of strategies for preventing misconduct.^3^^,^^5^^,^^7^^,^^8^ For example, if retractions were mostly due to plagiarism, it would be important to focus on procedures such as the use of automatic detection software and journal guidelines for handling plagiarism cases.^9^^,^^10^ On the other hand, for data fraud, more specific monitoring measures (for instance, introduction of data repositories, random audits and mandatory data sharing in an institution) would be appropriate.

## OBJECTIVE

Given the recent increase in retractions, this study aimed to characterize cases and reasons for scientific retractions in the field of general and internal medicine, in a high-profile international indexed database.

## METHODS

This study consisted of surveying the retraction notes in the Thomson-Reuters Web of Knowledge (WoK) indexing database,^11^ with special interest in the country of origin of the article and the impact factor (IF) of the journal in which the retraction was made. Articles classified as “general and internal medicine” were searched using the keywords “retraction” and “retracted” in their title fields (field tag = TI). After this initial identification, duplicate records and non-pertinent records of retraction (i.e. cases in which “retraction” referred, for instance, to surgical retraction) were removed, and the following information was gathered: country of origin of the article (main author address as defined by the indexing database); IF; country and name of the journal in which the retraction was made; year of publication of the retraction and the alleged reason for this (see below); and, finally, the journal and year in which the original work was published (in cases of plagiarism or duplication). The search procedures ended in November 2014.

The reasons for retraction were ascertained independently by three of the present authors and were classified as plagiarism or duplication, fraud or suspected fraud, error and authorship problems. The IF for the journal was obtained from the Journal Citation Reports database^12^ for the year closest to the retraction date. Additionally, the indicator “citations per paper” (CPP, i.e. the number of citations divided by the number of papers published over a specific period)^13^ was also obtained from the WoK database for the countries studied, covering the period from 2001 to 2011.^14^ When a CPP for a country was not available, its value was calculated directly from the WoK citation data with the aid of the “generate citation report” function, for the field of general and internal medicine.

The variables were aggregated as follows. The retraction reason was classified as “plagiarism/duplication” or “others”; the country of origin of the study was defined as high CPP or low CPP, dichotomized according to the median CPP value for the countries analyzed; and the IF was also dichotomized as high or low according to the median IF for the WoK-Web of Science subject area of general and internal medicine.^12^ Since the objective of the present work was basically descriptive, no modeling (e.g. logistic regression) was attempted. Instead, data were analyzed by means of cross-tabulating the retraction reasons against the aggregated country and IF for the periods 1992-2002 and 2003-2014. The relative risk of plagiarism/duplication was determined firstly according to the CPP (the proportion of retractions due to plagiarism in low-CPP countries divided by the proportion of retractions due to plagiarism in high-CPP countries); and secondly according to the IF (the proportion of retractions due to plagiarism in low-IF journals divided by the proportion of retractions due to plagiarism in high-IF journals). Following this, 95% confidence intervals (CIs) were then estimated for all retractions from 1992 to 2014. The data processing was performed using the SPSS version 2.0 software.

## RESULTS

Figure 1 shows the search strategy and number of retraction notes analyzed. After identification (through the title words “retraction of” and “retracted”) and screening (elimination of duplicate records and non-pertinent uses of the word “retraction”), a total of 86 notes were gathered. Out of these, the reasons for retraction could not be determined in six cases, which were not included in the analysis. These six “missing” cases came from journals published in Japan, Pakistan, South Korea and England. The first low-CPP/low-IF retraction case was seen in 1992 (due to plagiarism/duplication), and the next retraction note for the low-CPP/low-IF groups appeared in 2004 (also due to plagiarism/duplication).

**Figure 1.**
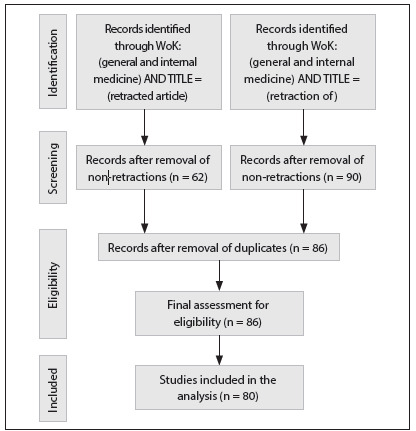
Search strategies and retraction notes identified, in relation to studies in the field of general and internal medicine, in the ISI Web of Knowledge (Wok) database.

CPP values were obtained directly for all countries except Croatia, Iran, Malaysia, Pakistan, Saudi Arabia and Tunisia, for which the manually calculated CPPs were 7.72, 6.97, 6.62, 7.58, 8.18 and 7.61, respectively. These countries, together with Brazil, China, India, South Korea, Taiwan and Turkey represented the “low-CPP” countries, while Belgium, Canada, Finland, Germany, Israel, Italy, Japan, Norway, Scotland, Switzerland, England and USA comprised the “high-CPP” group. Table 1 indicates that in the high-CPP countries, 12 retractions took place in 1992-2002, while in the low-CPP group, only one retraction was seen (due to plagiarism/duplication). However, over the period 2003-2014, these numbers were, respectively, 31 (high CPP) and 36 (low CPP). Overall, nearly one in six retractions in the high-CPP group were due to plagiarism/duplication, while in the low-CPP group, this proportion was much higher, resulting in a relative risk for plagiarism/duplication (RRplag-dup) of 3.4 (CI: 1.9-6.2). Thus, retractions due to plagiarism/duplication were 3.4 times more likely among low-CPP countries than among high-CPP countries. Similar results were seen for the high/low IF analysis, with a relative risk (RRIF) of 3.9 (CI: 2.0-7.8). The IF results for 1992-2002 are coincident with the CPP analysis for this period (not shown in Table 1).

**Table 1.**
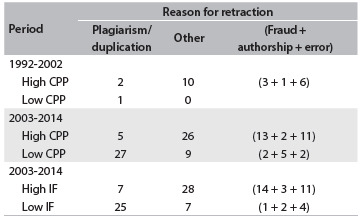
Retraction notes with identified reasons for retractions in the field of general and internal medicine, in the ISI Web of Knowledge database, aggregated according to countries of origin of the articles (high versus low, dichotomized according to the CPP median), impact factor (high versus low, dichotomized according to the IF median) and time period. The overall relative risk of plagiarism/duplication for the country group (reference group: high CPP), with 95% confidence interval, was 3.4 (1.9-6.2); for the IF group (reference: high IF), it was 3.9 (2.0-7.8). One case of mistaken duplication by an editor was classified as “error”

IF = impact factor; CPP = citations per paper.

## DISCUSSION

This study aimed to characterize retractions in the field of general and internal medicine, seen in a high-visibility indexing database, with a special interest in the reasons for retractions grouped according to the country of origin of the article country and the journal characteristics. Other studies (using broader databases such as the PubMed index)^5^^,^^7^^,^^8^ have also identified greater incidence of plagiarism among lower-income countries. However, for the present study, it was decided to use an indicator of scientific output as an alternative. Scientific misconduct is highly dependent on the scientific tradition and culture of a research group and, for strong scientific communities to be developed, generations of researchers need to be trained.^15^ Therefore, a well-known indicator of “scientific proficiency” was used: the number of citations per paper (CPP) of a country. This indicator is widely used for scientometric comparisons between countries^13^^,^^15^^,^^16^^,^^17^^,^^18^^,^^19^^,^^20^ and provides a means of measuring the research impact and visibility of a country.

It should also be noted that there is disagreement in the literature on this topic with regard to how the reasons for retractions should be grouped. For instance, plagiarism is sometimes regarded as a type of error, while other researchers have preferred to classify it together with duplication.^3^^,^^5^^,^^6^ In the present study, the latter option was adopted, given that: a) both of these types of misconduct involve inappropriate reporting and not flaws in experiments; b) the process of detecting them (e.g. using automatic detection software) is similar; and c) the measures for preventing them are similar. On the other hand, the lack of reporting on the reasons for retractions is a shortcoming that deserves attention from the scientific community, since, as mentioned earlier, precise characterization of the reasons that led to a retraction is a prerequisite for implementation of effective prevention strategies.

In the countries defined as low CPP, plagiarism/duplication accounted for a clear majority of the retraction cases, and a similar effect was seen in relation to the low-IF group (as expected, since most low-CPP cases were also low IF). Also in relation to the IF of the journal in which the retraction was made, some studies have pointed out that retractions are more common among high-IF journals,^21^ although this effect seemed to be leveling off in the more recent period analyzed here. One interpretation of these results is that, in countries with less tradition of research, procedures for ensuring academic integrity are also less widespread and, therefore, expansion of science in these countries leads to increases in the incidence of both retractions and plagiarism/duplication. In addition, detection of plagiarism has been made relatively easier by the internet and through the introduction of the aforementioned systems for automated detection.

Other results previously described in the literature could also be seen in the present study. For example, it is well established that retractions are a recent and increasing phenomenon,^2^^,^^6^ and this effect is even clearer if the low-CPP countries analyzed here are considered. In fact, in the present study, apart from one case that occurred in 1992, the first low-CPP/low-IF retraction note only appeared in 2004. In journals based in high-CPP countries, retractions have been present since 1992, but it is clear that they have recently been increasing.

The following limitations of the present study should be noted: a) the time periods used for estimating IFs and CPPs did not precisely correspond to the retraction dates; and b) the reasons for six retractions could not be ascertained. Another limitation relates to classification of reasons for retractions, which is not always straightforward and sometimes requires a “reading” of the reported information. However, in the present study, the researchers were in agreement regarding all the cases analyzed.

## CONCLUSION

It is well known that the frequency of scientific retractions has markedly increased over recent years. The present study documents the extent of this phenomenon among low-CPP countries in the field of general and internal medicine, using the WoK database. It found that plagiarism and duplication were the major cause of retractions among the countries involved, and similar results could be seen in relation to the low-IF journals in which the retractions were made. It is expected that studies such as the present one could lead to measures aimed towards international dissemination of best practices within research.
